# The Effects of High-Energy Carbon Co-Doping on IMB-CNM LGAD Fabrication and Performance

**DOI:** 10.3390/s25175571

**Published:** 2025-09-06

**Authors:** Jairo Villegas, Florent Dougados, Carmen Torres, Pablo Fernandez-Martinez, María del Carmen Jiménez-Ramos, Salvador Hidalgo

**Affiliations:** 1 Centro Nacional de Aceleradores (U. Sevilla, CSIC, J. de Andalucia), 41092 Sevilla, Spain; ctorres1@us.es (C.T.); mcyjr@us.es (M.d.C.J.-R.); 2 Instituto de Microelectrónica de Barcelona (IMB-CNM, CSIC), Universitat Autònoma de Barcelona, 08193 Barcelona, Spain; florent.dougados@imb-cnm.csic.es (F.D.); pablo.fernandez@imb-cnm.csic.es (P.F.-M.); salvador.hidalgo@csic.es (S.H.); 3 Departamento de Física Aplicada II, Universidad de Sevilla, 41012 Sevilla, Spain

**Keywords:** LGAD, X-rays and charged-particle detectors, alpha spectrometry, dopant diffusion in silicon

## Abstract

Over the past few years, Low-Gain Avalanche Detectors (LGADs) have demonstrated excellent timing performance, showing great potential for use in 4D tracking of high-energy charged particles. Carbon co-doping is a key factor for enhancing LGAD performance, which are detectors with intrinsic amplification, in harsh radiation environments. This work presents a broad pre-irradiation characterization of the latest carbon-co-implanted (or carbonated) LGADs fabricated at IMB-CNM. The results indicate that the addition of carbon reduces the nominal gain of the devices compared with non-carbonated detectors. Furthermore, a comprehensive study is presented on how carbon co-implantation can either enhance or suppress the diffusion of the multiplication layer during LGAD fabrication, depending on the device structure and fabrication parameters.

## 1. Introduction

**Figure 1:**
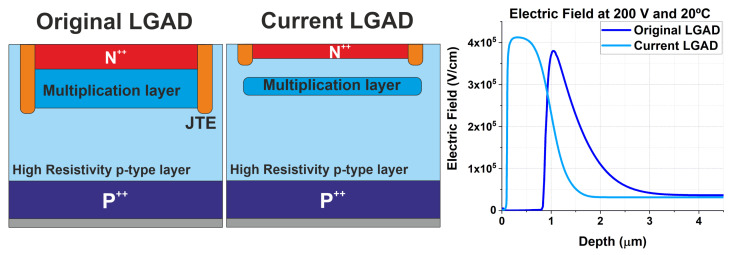
Schematics of the original and current IMB-CNM LGADs, along with their electric-field shapes, simulated with TCAD Sentaurus at 200 V and 20 °C.

Co-doping with carbon is a well-established technique in the fabrication of Low-Gain Avalanche Detectors (LGADs) to enhance their radiation tolerance. The original IMB-CNM LGADs [1] were fabricated by implanting carbon, boron (for the multiplication layer), and phosphorus (for the N electrode) at relatively low energies of ≈100 keV [2]. Recently, a new version of the LGAD has been developed at IMB-CNM, in which both carbon and boron are implanted at a higher energy of 480 keV, while arsenic, implanted at a low energy of 30 keV, is used for the N electrode. The technological differences between the original IMB-CNM LGADs and the current LGAD are schematically illustrated in Figure 1.

In the original LGADs, the doping profiles of the multiplication layer and the N-electrode overlap, and the resulting net charge distribution gives rise to the PN junction responsible for the high electric field that triggers avalanche multiplication. In contrast, in the current LGAD, the multiplication and N-electrode layers are physically separated by a high-resistivity silicon gap, allowing the formation of a quasi-flat high electric field within this gap, as shown in Figure 1.

This recent LGAD structure has been shown to exhibit both high uniformity in leakage current and outstanding timing performance before and after high levels of irradiation [3,4]. However, the effects of carbon co-doping at these high implantation energies on boron diffusion during fabrication are not yet fully understood. This work presents the main effects of carbon co-doping during LGAD fabrication and compares them to those observed in the original LGAD fabrication, which were comprehensively studied in [2].

## 2. LGAD Fabrication at IMB-CNM

Table 1 presents the main differences between the original LGADs [2] and the current IMB-CNM LGAD batch, which is fabricated on 150 mm high-resistivity epitaxial wafers with an active thickness of 55 μm. The fabrication parameters are presented in the table in a sequential manner.

On the one hand, the original LGADs are implanted with boron and carbon at higher doses and lower energies, followed by a primary annealing process of 180 min at 1100 °C to enable diffusion and activation. In contrast, for the recent LGADs, boron and carbon are implanted at lower doses but higher energies, and the layers undergo a low-temperature thermal process at 800 °C, which partially repairs the damage caused by the high-energy implantation. In both cases, an 84-min process at 950 °C is performed to grow a screen oxide prior to the implantation of phosphorus (original LGADs) or arsenic (current LGADs).

On the other hand, the original LGADs undergo long $N^{++}$ annealing for 30 min at 1000 °C, whereas the current LGADs are subjected to Rapid Thermal Annealing (RTA) for just one minute after arsenic implantation. In the latter case, RTA also serves to fully activate boron in the multiplication layer.

The current LGADs are fabricated with the specifications required for the Endcap Timing Layer (ETL) in the Compact Muon Solenoid (CMS) Phase-II upgrade at the High-Luminosity Large Hadron Collider (HL-LHC) [5], which aims to enhance the detection of minimum ionizing particles (MIPs) by improving the radiation tolerance and timing performance of the detectors. The latter is essential for mitigating pile-up from consecutive events and improving vertex reconstruction, which in turn enhances particle identification after proton–proton collisions. Specific applications within CMS include precision Higgs measurements, searches for new particles, and investigations beyond the Standard Model [5]. Furthermore, LGADs are also emerging as powerful tools beyond high-energy physics, with promising applications in fields such as space science and medical physics.

As previously mentioned, LGADs provide excellent timing performance, enabling precise time-of-flight measurements in space, thereby improving particle identification for cosmic-ray detection on satellites or space missions [6]. In medicine, LGADs are being explored for beam monitoring in FLASH radiation therapy [6], where their gain and timing precision allow real-time tracking of therapeutic particle beams. This ensures accurate dose delivery and enhances patient safety during ultra-short, high-intensity radiation treatments. For both applications, the enhanced radiation tolerance of LGADs over conventional silicon detectors is essential for reliable operation under harsh radiation environments.

In this work, single-pad LGAD samples (current IMB-CNM version) with an active area of 1.3 × 1.3 mm^2^ were studied.

## 3. The Role of Carbon Co-Doping During LGAD Fabrication

As presented in Table 1, a key difference between the fabrication of the original and current IMB-CNM LGADs lies in the annealing and activation times for the 1100 °C thermal step. The original LGADs undergo much longer annealing at this temperature to achieve proper electrical balance and overlap between the n- and p-type silicon around the PN junction. In contrast, the current LGADs are designed with a much shorter annealing duration (30 s) to activate boron and partially repair implantation damage while minimizing dopant diffusion. In both cases, the presence of carbon co-doping significantly affects the diffusion of implanted dopants and, consequently, the electrical performance of the devices.

The main effect of carbon co-doping in the original IMB-CNM LGADs was comprehensively studied in [2]. The primary consequence of adding carbon to this structure is an increased gain response at a given bias voltage compared to an original LGAD fabricated under the same conditions without carbon. This improvement is attributed to carbon atoms blocking excess interstitial paths, which slow the diffusion of both boron and phosphorus by suppressing transient enhanced diffusion (TED) through these paths [2,7,8].

However, in the recent LGADs, the high-energy boron and carbon implants cause significant lattice damage and generate an excess of interstitials, which promote TED of boron. Although carbon can trap interstitials and eventually suppress diffusion, the initial burst of interstitials during 800 °C annealing can still enhance boron diffusion before suppression takes effect [9].

**Figure 2:**
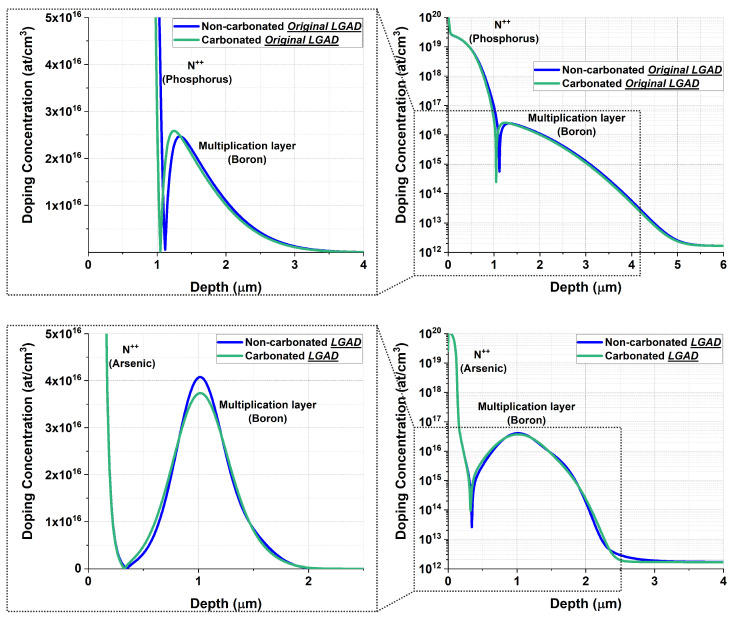
TCAD Sentaurus simulations of the doping profiles for the original IMB-CNM LGAD structure (**top**) and the current LGAD structure (**bottom**), both with and without carbon co-implantation. The images on the (**left**) show the profiles on a linear scale, while those on the (**right**) use a logarithmic scale to better distinguish the N-electrode profiles.

In short, the presence of carbon in the structures of either the original or current IMB-CNM LGADs does not instantly suppress boron diffusion. Its suppressive effect depends on carbon interstitial trapping, which competes with TED during the early stages of annealing. When TED dominates initially, as in the case of the current LGADs, boron can diffuse further before enough interstitials are trapped to slow the process. As a result, the doping peak in the multiplication layer is lower in a carbonated LGAD, leading to a reduced peak electric field compared to a non-carbonated LGAD. In contrast, if the annealing time is sufficiently long for carbon atoms to occupy interstitial sites, TED can be effectively suppressed. In this case, a carbonated original IMB-CNM LGAD exhibits a higher boron peak in the multiplication layer, resulting in a stronger electric field [2]. The aforementioned effects were studied using TCAD Sentaurus (v.2018.06; Synopsys, Inc; Sunnyvale, CA, USA) simulations [10]. Figure 2 shows the simulated doping profiles for both the original and current IMB-CNM LGAD structures, both with and without carbon co-implantation, using the fabrication parameters listed in Table 1. For the original LGAD, a carbon implantation dose of 4 · $10^{14}$ at/cm^2^ was used. The simulation results indicate that the effects of carbon on the doping peak of the multiplication layer are the opposite for the original and current IMB-CNM LGAD structures.

**Figure 3:**
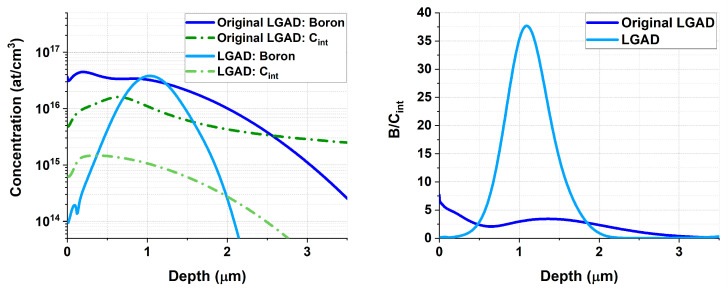
TCAD Sentaurus simulations of the boron and interstitial carbon ($C_{int}$) concentrations (**left**) for the original and current IMB-CNM LGAD structures, along with the ${B/C}_{int}$ ratios (**right**) for these devices.

A deeper understanding of this effect was provided by TCAD Sentaurus simulations of interstitial carbon ($C_{int}$) concentrations for both an original and a current IMB-CNM LGAD structure, following the fabrication process described in Table 1. These results are shown in Figure 3, where the image on the right displays the ratio between the concentration of boron (multiplication layer) and interstitial carbon. For the recent LGAD, this ratio is significantly higher, indicating that fewer interstitial diffusion paths are being blocked by carbon atoms.

## 4. Electrical Characterization of LGAD Samples at IMB-CNM

Capacitance and leakage current vs. bias voltage measurements were carried out on 15 LGAD samples per device type (non-carbonated, or simply LGAD, and carbonated, denoted as c.LGAD). The CV curves were measured using a Keysight Agilent 4284A LCR meter (Keysight Technologies, Santa Rosa, CA, USA) in series mode at 20 °C, 10 kHz, and 200 mV AC. The IV curves were obtained at 20 °C and −25 °C with a Keithley 4210 (Keithley Instruments, Cleveland, OH, USA). In both cases, the guard ring (Figure 1) was connected to separate the surface current from the bulk current. IV measurements were conducted at both room temperature (20 °C) and −25 °C, the latter being the expected operating temperature for the CMS ETL Phase-II upgrade for which the fabricated LGADs were designed [5].

**Figure 4:**
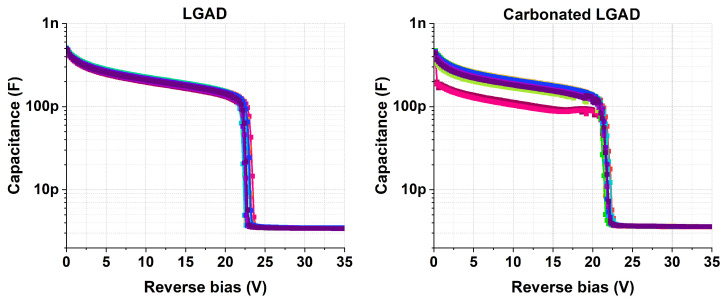
CV curves, at 20 °C, 10 kHz, 200 mV AC and series mode, for 15 LGAD and 15 c.LGAD samples.

**Figure 5:**
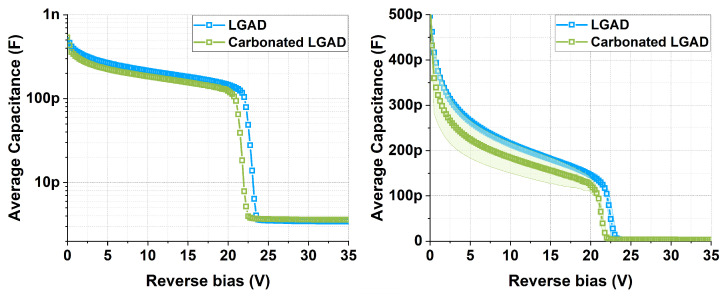
Average capacitance at 20 °C, 10 kHz, 200 mV AC, series mode, and with standard deviation bands for the studied samples. Both images show the same data using different scales: logarithmic on the (**left**) and linear with standard deviation bands on the (**right**).

Figure 4 shows the CV curves for the studied detectors. The results show that the c.LGADs exhibit greater capacitance dispersion before reaching the depletion voltage of the multiplication layer ($V_{gl}$). This is more clearly observed in Figure 5, which also shows that the average $V_{gl}$ is higher for the non-carbonated samples, with values of $V_{gl}\left(LGAD\right)=21.5$ and $V_{gl}\left(c.LGAD\right)=20.4$. These $V_{gl}$ values were calculated using the methodology described in [2].

**Figure 6:**
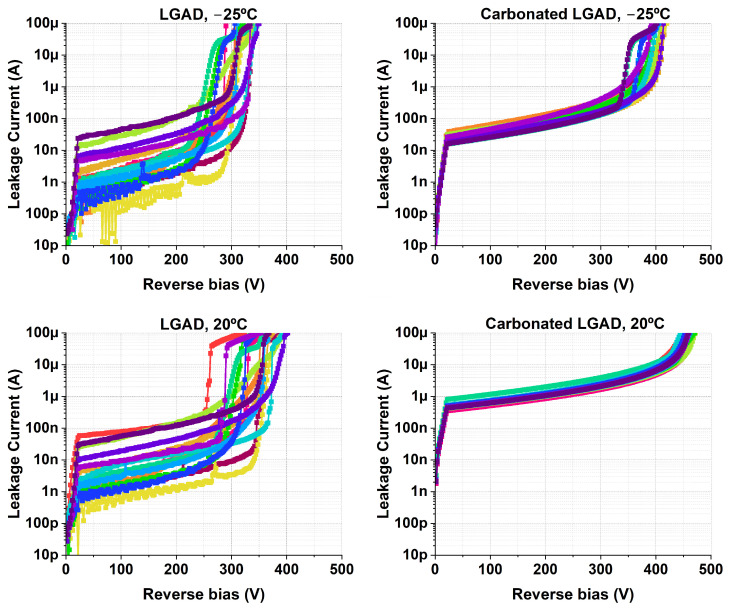
IV curves at −25 °C and 20 °C for 15 LGAD samples and 15 c.LGAD samples.

**Figure 7:**
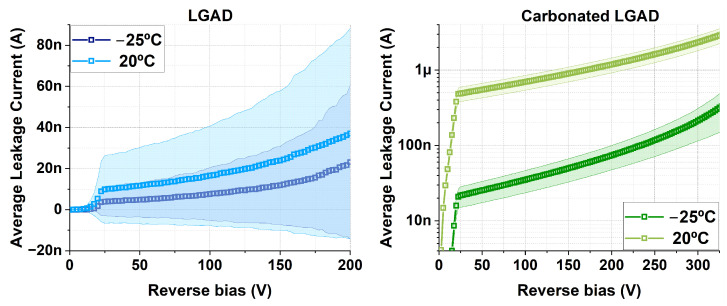
Average leakage current with standard deviation bands for the studied samples (LGAD on the (**left**), c.LGADs on the (**right**)). For the c.LGADs, the results are shown on a logarithmic scale to better distinguish the curves at −25 °C and 20 °C.

The IV curves at 20 °C and −25 °C are shown in Figure 6. On the one hand, the results indicate that the c.LGADs exhibit both a higher leakage current and a higher breakdown voltage, independent of the measurement temperature. On the other hand, the leakage current is also more uniform in the carbonated samples. This effect is better depicted in Figure 7, where the average leakage current with standard deviation bands is shown up to the bias voltage at which the first device breaks down.

Although the leakage current increases from −25 °C to 20 °C in both LGADs, the standard deviation is so large for the non-carbonated samples that their error bands overlap. In contrast, c.LGADs show a higher but more uniform leakage current, with clearly distinguishable behavior between −25 °C and 20 °C. Notably, the higher leakage current observed in the c.LGADs is consistent with previous findings reported in [4].

Overall, the CV and IV measurements show that the non-carbonated LGADs exhibit a higher depletion voltage of the multiplication layer ($V_{gl}$) but a lower breakdown voltage compared to the carbonated samples. This behavior is consistent with the presence of a sharper doping concentration peak in the multiplication layer [2], supporting the hypothesis discussed in Section 3. Furthermore, the non-carbonated devices exhibit a lower leakage current but show greater variation across samples compared to their carbonated counterparts. However, this higher dispersion does not seem to originate from variations in the shape of the multiplication layer, as it is not reflected in the more uniform CV behavior observed for these devices (Figure 4 and Figure 5).

In contrast, the c.LGADs show a higher but more uniform leakage current across samples, but their capacitance values before reaching $V_{gl}$ are less uniform. While these phenomena are likely linked to ion implantation-induced defects, their influence on the electrical behavior may depend on specific defect characteristics, such as concentration, spatial distribution, and energy levels within the bandgap. Hence, a more comprehensive understanding will require further studies with increased sample statistics and detailed defect characterization.

**Figure 8:**
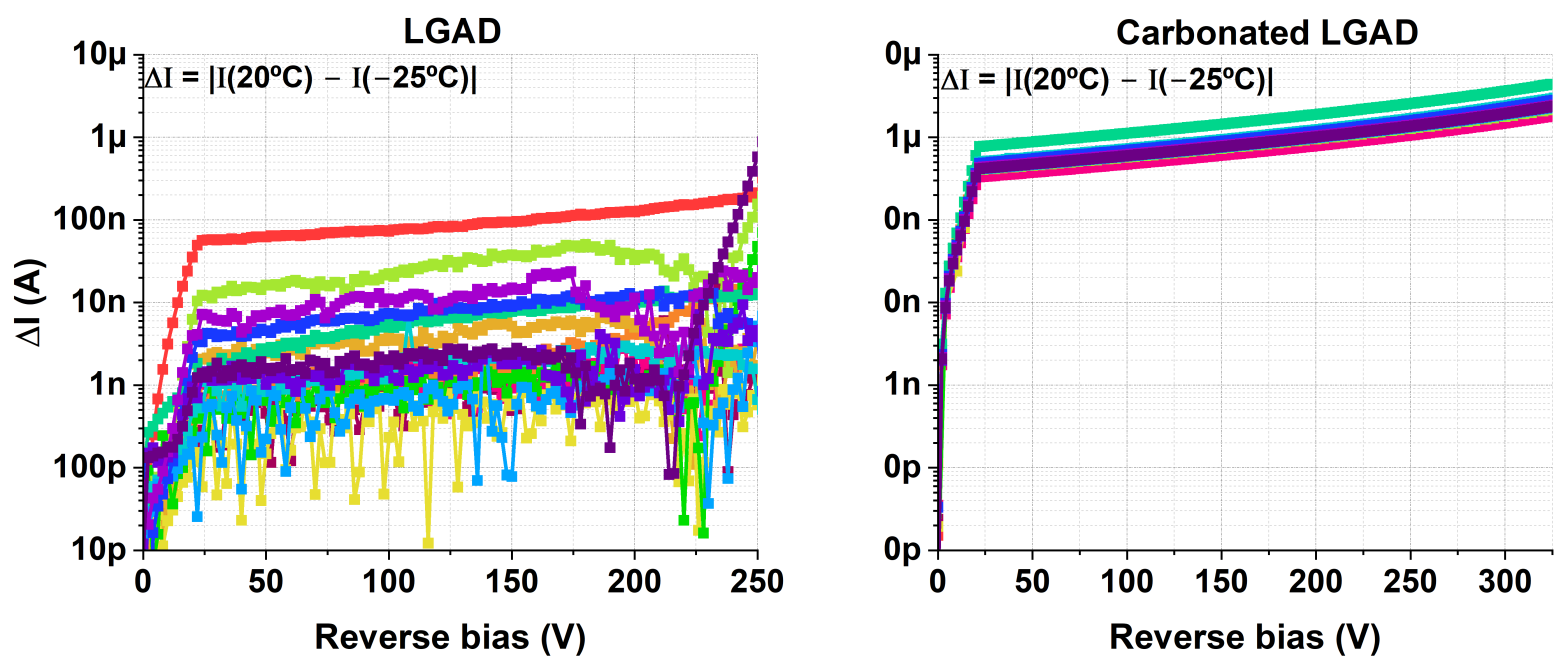
ΔI = |I(20 °C) − I(−25 °C)| for the studied LGAD and c.LGAD devices, evaluated up to the bias voltage at which the first device of each type breaks down.

A preliminary hypothesis is that the excess implantation-induced defects from carbon co-implantation may be responsible. If carbon co-implantation leads to an oversaturation of defects, then the leakage current would saturate at the elevated value reported. In contrast, without carbon, the smaller number of boron implantation defects may be distributed more non-uniformly across the wafer, which could explain the observed sample-to-sample variations. An initial step to support this hypothesis is the evaluation of ΔI = |I(20 °C) − I( −25 °C)| for each device, as shown in Figure 8. The leakage current of an LGAD can be expressed as [11,12]

(1) $$I\propto f_{1}\left(G\right)f_{2}\left(T\right)\Sigma N_{def}\left(T\right)$$

where $f_{1}\left(G\right)$ and $f_{2}\left(T\right)$ are gain- and temperature-dependent functions, respectively, and $N_{def}\left(T\right)$ is the number of defects in the silicon lattice, whose temperature dependence varies with the defect type. With uniform gain and defect density across the wafer, ΔI should remain constant across devices, as observed for the c.LGADs in Figure 8, where most of the curves overlap. In contrast, ΔI shows a larger dispersion for the non-carbonated LGADs. According to Equation (1), this dispersion may arise from variations in either the gain or the number of defects from sample to sample. Nevertheless, additional testing with appropriate methods is necessary to verify this hypothesis. Techniques like Deep-Level Transient Spectroscopy (DLTS) [13] may provide valuable insight into variations in defect density across different samples.

It is worth remarking that the Keithley 4210 used to obtain the IV curves has a precision of ∼1 nA, which explains why ΔI exhibits apparent oscillating noise for values below this threshold in Figure 8.

## 5. LGAD Gain Measurements to Alpha Particles at CNA

**Figure 9:**
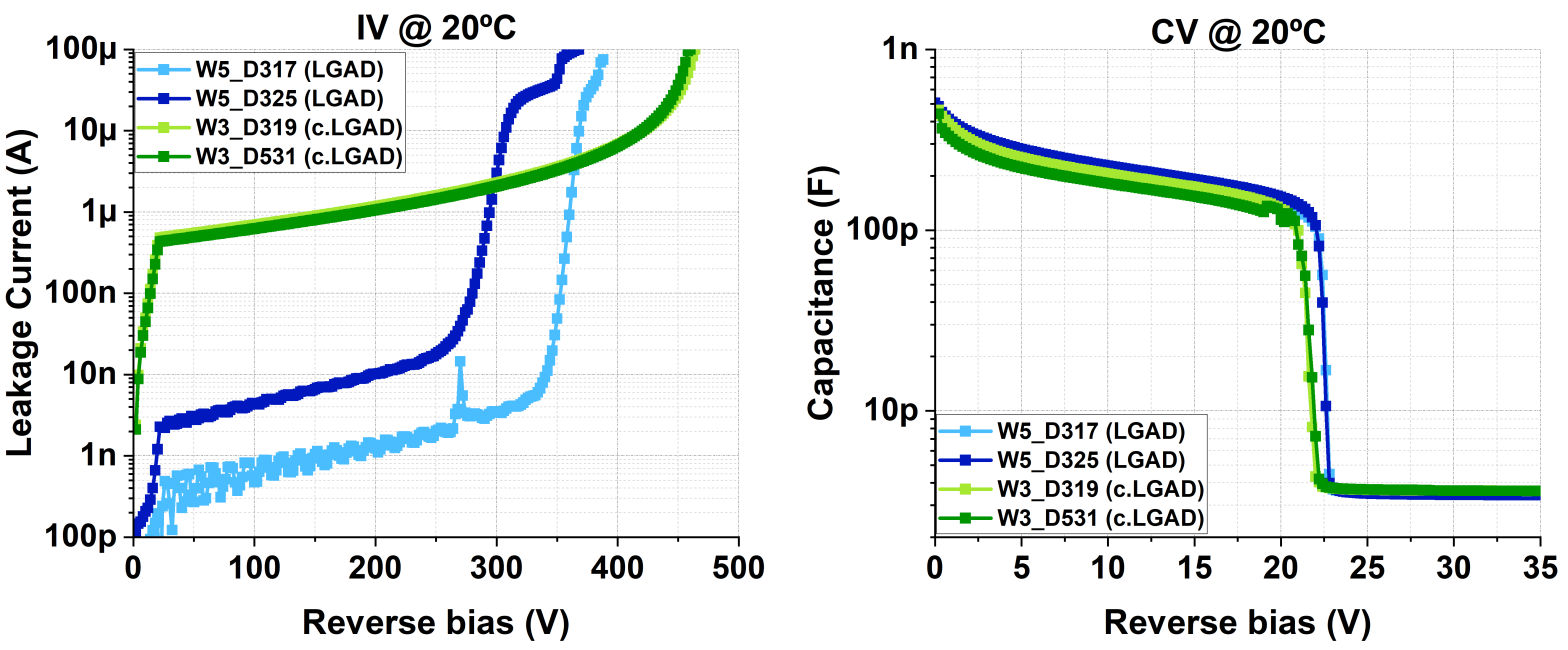
IV and CV curves of the tested LGADs, where c.LGAD denotes the carbonated devices.

Four LGAD devices, with and without carbon co-implantation, were studied for their gain to alpha particles. Their IV and CV curves are shown in Figure 9. A triple-alpha source containing ^239^Pu, ^241^Am, and ^244^Cm (emitting alpha particles with energies of 5.15, 5.48, and 5.79 MeV, respectively) was used for this purpose. Each isotope had the same activity of 3 kBq. A reference PiN detector was also studied to extract the gain.

**Figure 10:**
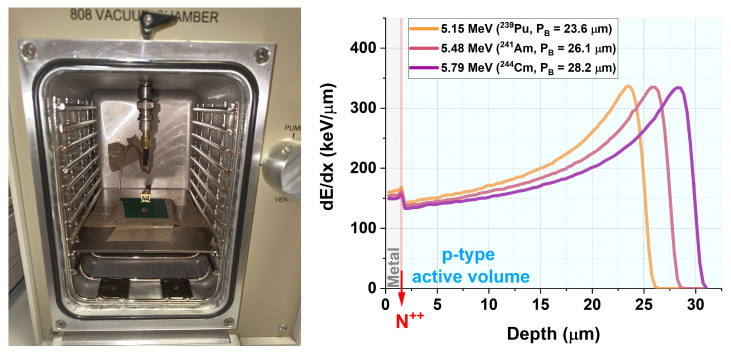
(**Left**) The vacuum chamber. A green portable circuit board (PCB) with a mounted device is also shown. (**Right**) SRIM simulation of the alpha particles’ energy loss within the studied detectors.

Signal acquisition and subsequent processing were performed using a conventional electronic chain consisting of a high-voltage Keithley 2470 source (Keithley Instruments, Cleveland, OH, USA), a CANBERRA preamplifier model 2003BT (Mirion Technologies, Alpharetta, GA, USA) with a modified impedance of R = 10 MΩ, a CANBERRA amplifier model 2022 (Mirion Technologies, Alpharetta, GA, USA), and an AMPTEK multi-channel analyzer model 8000D (AMPTEK Inc., Bedford, MA, USA). The setup was enclosed in a vacuum chamber to prevent energy loss of the alpha particles in air, as shown in Figure 10.

The detectors and sources were kept at a fixed distance to ensure reproducible measurement conditions. This distance was maintained by placing them on the internal support shelves of the vacuum chamber, as shown in Figure 10. The vacuum chamber was evacuated using a mechanical pump for a few minutes prior to each measurement to minimize the presence of residual air. This procedure ensured stable operation and minimized the energy loss of the alpha particles through air.

As the thickness and material composition of the dead entrance window layers were known, the alpha-particle energies after traversing these layers could be determined using SRIM (Stopping Range of Ions in Matter) simulations [14]. These layers consisted of 1.5 μm of aluminum, 300 Å of titanium, and ≈0.2 μm of the non-depleted N electrode. While the thicknesses of the metallic layers were measured during fabrication, the N-electrode thickness was estimated using both TCAD Sentaurus simulations and reverse engineering of other IMB-CNM detectors with an $N^{++}$ electrode fabricated with the same parameters listed in Table 1.

With this data, the corrected alpha energies impinging on the active volume of the detector were 4.87 ± 0.01 MeV (^239^Pu), 5.21 ± 0.01 MeV (^241^Am), and 5.53 ± 0.02 MeV (^244^Cm). The uncertainties represent the standard deviation of the average alpha-particle energies after passing through the dead entrance window. Figure 10 shows the SRIM-simulated energy deposition within the detector, demonstrating that the alpha particles of these energies were fully absorbed within the device active volume at Bragg peak depths ($P_{B}$) ranging from 20 to 30 μm. It is worth noting that gain-suppression effects are reported for ions that produce high ionization charge densities within the detector’s active volume [15,16], such as the alpha particles studied in this work.

**Figure 11:**
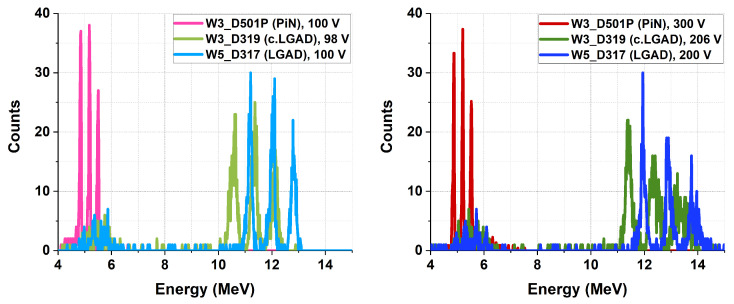
(**Left**) Tri-alpha spectra for the reference PiN (100 V), LGAD (100 V), and c.LGAD (98 V). (**Right**) Tri-alpha spectra for the reference PiN (300 V), LGAD (200 V), and c.LGAD (206 V).

With the deposited alpha energies known, the multichannel analyzer was calibrated in energy using the reference PiN detector. Figure 11 shows some of the spectra for the PiN, LGAD, and carbonated LGAD (c.LGAD) after calibration. The results indicate that the PiN peaks remained constant with bias, showing no shift as the voltage increased from 150 V to 300 V. In contrast, both the LGAD and c.LGAD peaks shifted to higher energy values as the bias increased due to their internal multiplication mechanism.

**Figure 12:**
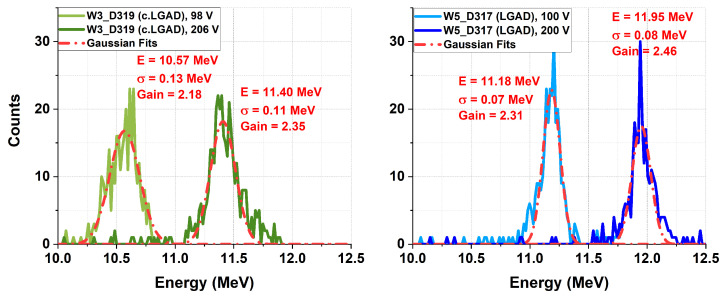
Tri-alpha spectra of the ^239^Pu source (5.15 MeV alpha particles) for the LGAD at 100 V and 200 V, and for the c.LGAD at 98 V and 206 V.

The gain was then extracted as the ratio of the energy measured in the LGAD to that measured in the PiN, which is equivalent to the ratio of the charges collected by the detectors. The measured energy is defined as the centroid obtained from a Gaussian fit, as shown in Figure 12. The centroid uncertainty is statistically inferred by dividing the sigma of the Gaussian fit by the square root of the number of counts within its energy range. For all studied devices, the uncertainty associated with the fit was ∼1 %.

For the c.LGADs, with a leakage current I on the order of μA, the actual voltage applied to the detector was corrected as $V_{keithley}$–IR due to the voltage drop across the preamplifier impedance. For the non-carbonated LGADs, such a correction was not necessary, as their low leakage current resulted in a voltage drop of less than 1 V.

**Figure 13:**
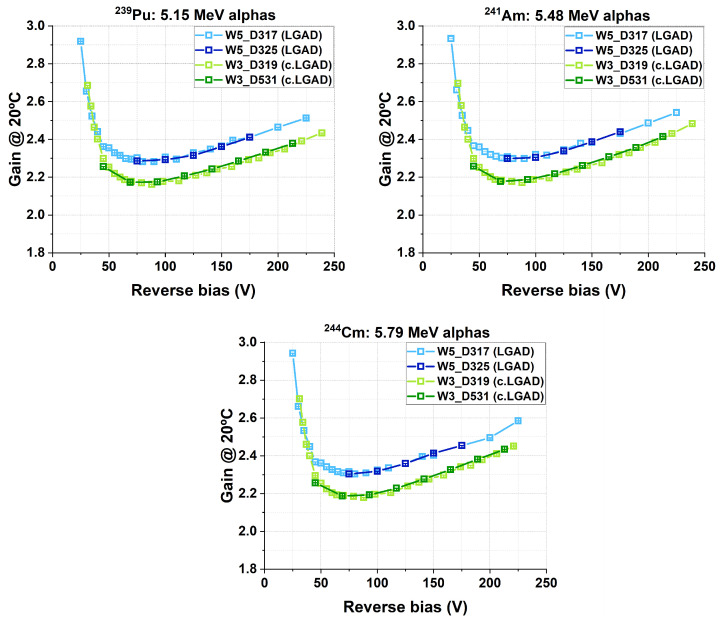
Gain results for 5.15, 5.48, and 5.79 MeV alpha particles for the studied detectors.

Figure 13 shows the gain results extracted using the methodology described above. In the graph, error bars are included but not distinguishable due to their very small size. The results indicate that the gain response of detectors from the same wafer was highly uniform across samples. Furthermore, they suggest that the non-uniform leakage current observed in the non-carbonated devices (Figure 9) did not significantly affect the gain, as similar gain values were obtained despite different leakage currents. Finally, the c.LGADs exhibited a lower gain than their non-carbonated counterparts, a result consistently observed across all three alpha energies.

It is worth noting that the observed gain behavior, where the gain reaches a maximum after full depletion and subsequently never returns to the same value, has already been reported in the context of alpha-particle detection with LGADs. A comprehensive review of this phenomenon can be found in [16].

**Figure 14:**
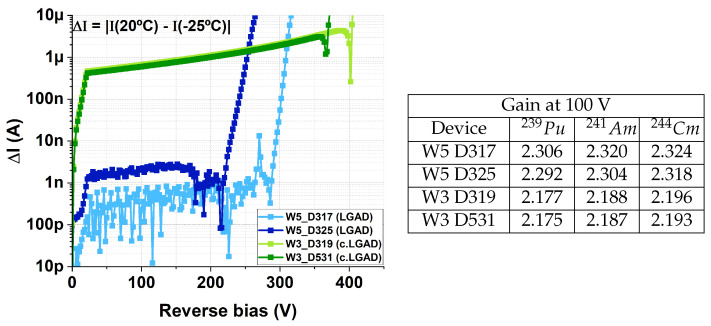
ΔI = |I(20 °C) − I(−25 °C)| for the studied LGAD and c.LGAD devices, shown alongside their gains at 100 V for different alpha-particle sources.

On the other hand, the results shown in Figure 13 reflect the uniformity of the gains for LGAD and c.LGAD devices. This indicates that the differences in ΔI observed in Figure 8 were not due to variations in gain across different samples (Equation (1)). Figure 14 shows ΔI for the studied detectors, highlighting that, for the non-carbonated devices, the observed variations are not related to the gain. This result opens the door to further investigate whether variations in the density of implantation-induced defects, in the absence of carbon co-doping, may be responsible for the leakage current dispersion reported in the previous section.

**Figure 15:**
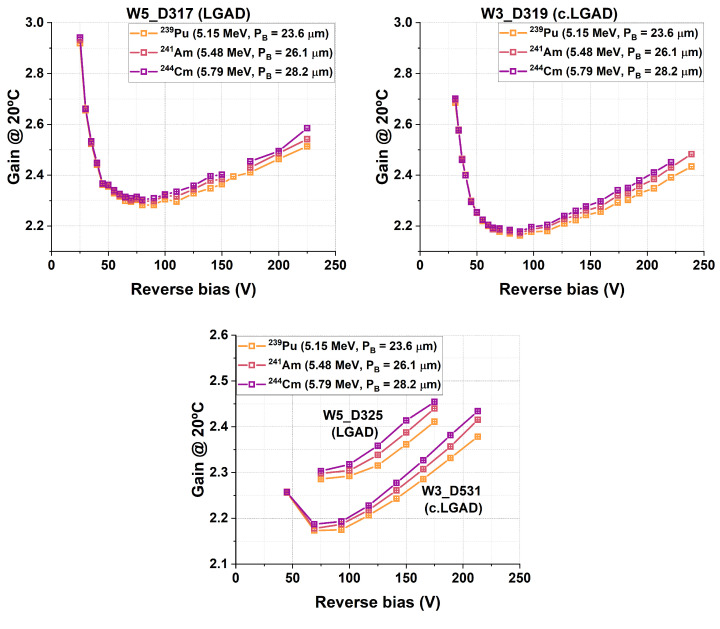
Gain results for 5.15, 5.48, and 5.79 MeV alpha particles for the studied detectors. In contrast to Figure 13, the results in each graph are presented for individual detectors, highlighting the reduction in gain-suppression effects with increasing alpha energy.

**Figure 16:**
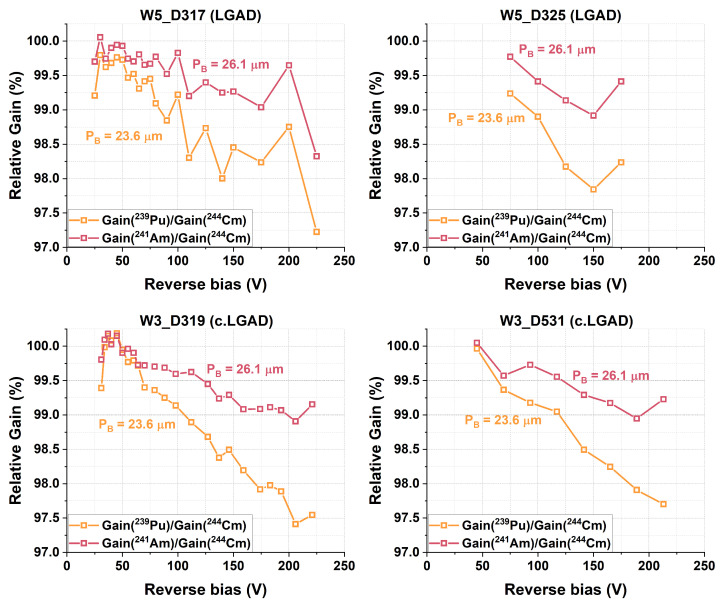
Relative gain with respect to the alpha particle with the highest $P_{B}$ value of 28.2 μm (5.79 MeV alphas from the ^244^Cm source) for the studied LGAD and c.LGAD devices.

A further analysis of the gain as a function of the alpha energy (Figure 15) revealed that gain-suppression effects were consistently, albeit slightly, less pronounced for higher energy values. This result is consistent with previous findings, which indicate that the depth of the Bragg peak influences gain suppression: the closer the Bragg peak ($P_{B}$) is to the PN junction, the lower the gain response for a given particle species [15]. This effect is better visualized in Figure 16, where the relative gain with respect to the alpha particle with the highest $P_{B}$ value of 28.2 μm (5.79 MeV alpha particles from the ^244^Cm source) is shown for each device. The results show that the gain was consistently more strongly suppressed for the alpha particle from ^239^Pu, which had the lowest energy (5.15 MeV) and $P_{B}$ value (23.6 μm).

Furthermore, gain suppression may also arise from differences in the multiplication layer profile between devices with and without carbon co-implantation. Previous studies have shown that gain is more strongly suppressed in devices that initially have a higher unsuppressed gain than those with lower gain [17]. In other words, a device with a higher multiplication layer peak (e.g., LGAD), which corresponds to a higher electric field and gain at a given bias, experiences stronger gain suppression than a device with a lower peak (e.g., c.LGAD). Understanding the relationship between the local electric field and gain suppression will require future studies with LGADs fabricated using different multiplication layer designs and measurements using other particle species for comparison.

It is worth noting that the studied LGADs are intended for detecting MIPs both before and after irradiation at fluences on the order of $10^{15}$/cm^2^ (1 MeV neutron-equivalent) [5]. While gain studies with low-penetration alpha particles have provided an initial assessment of device performance and its relationship to the technological effects of carbon co-implantation, further investigations of gain with MIPs (both before and after irradiation) are required to fully evaluate the viability of the current IMB-CNM LGADs for their intended application.

## 6. Conclusions

The studies presented in this work demonstrate that the effects of carbon co-implantation on the electrical performance of LGADs may differ from those observed in the original IMB-CNM LGADs. Dopant diffusion in the presence of carbon appears to have opposing effects in the original and current IMB-CNM LGAD structures. In the latter case, carbonated devices exhibit a lower $V_{gl}$ and gain response, along with a higher breakdown voltage, as shown in Table 2.

Conversely, in the original LGAD structure, carbonated devices show a higher $V_{gl}$ and gain response, as well as a lower breakdown voltage [2].

This dopant diffusion effect has also been correlated (via TCAD Sentaurus simulations) with the number of implanted carbon atoms occupying interstitial sites in the silicon lattice after annealing and activation of the device layers. Notably, alternative annealing strategies, such as annealing the carbon layer prior to boron implantation for the multiplication layer, may influence the number of interstitial sites occupied by carbon atoms. A pre-annealing step may promote the stabilization of carbon atoms in interstitial positions before the boron implant, thereby increasing the likelihood of carbon effectively suppressing boron TED, as observed in the original LGAD. In turn, this could result in a sharpening of the multiplication layer and modify the impact of carbon co-doping on the electrical performance of LGADs during fabrication.

Additionally, the analysis of the leakage current at 20 °C and −25 °C suggests that the dispersion in the leakage current for the non-carbonated devices may result from greater non-uniformity in implantation-induced defect density across the wafer. However, this hypothesis requires further validation using viable techniques, such as DLTS, which may help determine the defect density from sample to sample.

## Figures and Tables

**Table 1:** Main fabrication parameters of the original and current IMB-CNM LGADs.

	Original LGAD	Current LGAD
Carbon dose (at/cm^2^)	$10^{14}$–9 × $10^{14}$	5 × $10^{13}$
Carbon energy (keV)	150	480
Boron dose (at/cm^2^)	1.9 × $10^{13}$	2.5 × $10^{12}$
Boron energy (keV)	100	480
Carbon and multiplication layer annealing	180 min @ 1100 °C + 84 min @ 950 °C	104 min @ 800 °C + 84 min @ 950 °C
Phosphorus dose (at/cm^2^)	5 × $10^{14}$ & $10^{15}$	-
Phosphorus energy (keV)	70 & 150	-
Arsenic dose (at/cm^2^)	-	$10^{15}$
Arsenic energy (keV)	-	30
$N^{++}$ layer annealing	30 min @ 1000 °C	RTA: 30 s @ 1100 °C + 30 s @ 1050 °C

**Table 2:** Main electrical parameters obtained for the studied LGAD and c.LGAD devices.

	LGAD	c.LGAD
$V_{gl}$ (V)	21.5	20.4
Breakdown Voltage at −25 °C (V)	250–325	350–400
Average Gain at 100 V (^239^*Pu*)	2.30	2.18
Average Gain at 100 V (^241^*Am*)	2.31	2.19
Average Gain at 100 V (^244^*Cm*)	2.32	2.20

## Data Availability

The dataset is available on request from the authors.

## Abbreviations

The following abbreviations are used in this manuscript:

AC	Alternating Current
$C_{int}$	Interstitial Carbon
c.LGAD	Carbonated Low-Gain Avalanche Detector
CMS	Compact Muon Solenoid
CNA	Centro Nacional de Aceleradores
CV	Capacitance vs. Voltage
DLTS	Deep-Level Transient Spectroscopy
ETL	Endcap Timing Layer
IMB-CNM	Instituto de Microelectrónica de Barcelona-Centro Nacional de Microelectrónica
IV	Leakage Current vs. Voltage
LGAD	Low-Gain Avalanche Detector
LRC	Inductor Resistor Capacitor
MIP	Minimum Ionizing Particle
PiN	P-type–intrinsic-N-type Detector
PN	P-type–N-type
RTA	Rapid Thermal Annealing
SRIM	Stopping Power of Ions in Matter
TCAD	Technology Computer-Aided Design
TED	Transient Enhanced Diffusion
$V_{gl}$	Depletion Voltage of the Multiplication Layer
